# A fast and reliable polymerase chain reaction method based on short interspersed nuclear elements detection for the discrimination of buffalo, cattle, goat, and sheep species in dairy products

**DOI:** 10.5713/ajas.18.0459

**Published:** 2019-01-02

**Authors:** Gianfranco Cosenza, Marco Iannaccone, Daniela Gallo, Alfredo Pauciullo

**Affiliations:** 1 Department of Agricultural Sciences, University of Naples “Federico II”, Portici, NA 80055, Italy; 2 Department of Agricultural, Forest and Food Science, University of Torino, Grugliasco, TO 10095, Italy

**Keywords:** DNA Detection, Short Interspersed Nuclear Elements (SINE), Polymerase Chain Reaction (PCR), Dairy Products, Ruminants

## Abstract

**Objective:**

Aim of present study was the set up of a fast and reliable protocol using species-specific markers for the quali-quantitative analysis of DNA and the detection of ruminant biological components in dairy products. For this purpose, the promoter of the gene coding for the α-lactoalbumin (*LALBA*) was chosen as possible candidate for the presence of short interspersed nuclear elements (SINEs).

**Methods:**

DNA was isolated from somatic cells of 120 individual milk samples of cattle (30), Mediterranean river buffalo (30), goat (30), and sheep (30) and the gene promoter region (about 600/700 bp) of *LALBA* (from about 600 bp upstream of exon 1) has been sequenced. For the development of a single polymerase chain reaction (PCR) protocol that allows the simultaneous identification of DNA from the four species of ruminants, the following internal primers pair were used: 5′-CACTGATCTTAAAGCTCAGGTT-3′ (forward) and 5′-TCAGA GTAGGCCACAGAAG-3′ (reverse).

**Results:**

Sequencing results of *LALBA* gene promoter region confirmed the presence of SINEs as monomorphic “within” and variable in size “among” the selected species. Amplicon lengths were 582 bp in cattle, 592 bp in buffalo, 655 in goat and 729 bp in sheep. PCR specificity was demonstrated by the detection of trace amounts of species-specific DNA from mixed sources (0.25 ng/μL).

**Conclusion:**

We developed a rapid PCR protocol for the quali-quantitative analysis of DNA and the traceability of dairy products using a species-specific marker with only one pair of primers. Our results validate the proposed technique as a suitable tool for a simple and inexpensive (economic) detection of animal origin components in foodstuffs.

## INTRODUCTION

Food safety and quality have become increasingly important world-wide following the bovine spongiform encephalopathy outbreak in 1992, when the role of infected animal products for spreading transmissible animal diseases was brought to light. To protect the consumer’s health, but also to meet requirements from international trade, a major concern to food safety regulation is the ability to demonstrate the origin and the authenticity of food products. Indeed, Regulation EC/178/2002 defines traceability as the “ability to trace and follow food, feed, and ingredients through all stages of production, processing and distribution”. This means that we should be able to trace our food from “farm to the fork” and track it back too. The traceability systems are the principal tool to both ensure the effective responsibility of food manufacturers, farmers and food operators in relation to the final product quality, and at the same moment to assess and manage risks effectively [1]. Therefore, the “farm to the fork” approach is a key strategy to fight food adulteration in relation to the current food safety requirements.

In this context, buffalo milk adulteration is one the most common type of food manipulations. Buffalo milk is characterised by a higher level of fat (on average 8.0%) and proteins (on average 4.5%) compared to the milk of other ruminants [2] and it is almost completely processed into buffalo Mozzarella cheese, a typical Italian product certificated by the European Protected Designation of Origin (PDO) (EC Regulation No 1107/96 of 12 June 1996). Considering the growing market demand for Mozzarella PDO and a higher profit obtainable by producers and sellers, the fraudulent addition of poorer and cheaper ruminant milk during the dairy manufacturing has become an alarming illegal activity.

Currently, several analytical procedures are available for species identification in milk products (EC Regulation No. 213/2001) including protein gel electrophoresis [3], immunochemistry [4], chromatography [5], and mass spectrometry [6]. However, one of the main disadvantages of these techniques is their application only on fresh products, because of the protein’s degradation after cooking process.

Several techniques based on DNA detection such as classic polymerase chain reaction (PCR) [7], duplex-PCR [8], PCR-restriction fragment length polymorphism [9] and quantitative Real Time PCR [10,11] were developed to distinguish the presence of DNA contaminations from other species in buffalo milk samples. However, these methods require different amplification reactions using different primer pairs or additional processing steps, such as restriction endonuclease digestion or hybridization for scoring. Therefore, they are laborious and time-consuming.

The goal of this study was to develop a rapid PCR protocol for the quali-quantitative analysis of DNA, for the traceability of dairy products using a species-specific marker with only one pair of primers. Among the numerous markers useful for this purpose, the short interspersed nuclear elements (SINEs) are particularly suitable. In fact, SINE retrotransposition events have proven their value as phylogenetic markers in several eukaryotic taxa at different taxonomic levels, clustering all species or subspecies that share a specific insertion [12,13]. Thus, using a single pair of primers in a single reaction, we amplified a SINE element in the promoter region of the α-lactoalbumin (*LALBA*) encoding gene, suggested as useful phylogenetic markers in the ruminant families, subfamilies and tribes [12].

In this way, we developed for the first time a method for the simultaneous detection of buffalo, cattle, goat, and sheep DNA in dairy products.

## MATERIALS AND METHODS

### Samples and DNA extraction

In this study no animals were slaughtered. The experimental plan was performed according to Directive 2010/63/EU of the European Parliament (European Union, 2010) and Directive 86/609/EEC (European Economic Community, 1986), which deal with the protection of animals used for scientific purposes.

DNA was isolated from somatic cells, recovered from 120 individual milk samples of cattle (30), Mediterranean river buffalo (30), goat (30), and sheep (30) reared in Italy collected by hand-milking after udder cleaning from different breeds/genetic types randomly chosen, using the procedure described by Pokorska et al [14]. DNA concentration and optical density_260/280_ (OD_260/280_) ratio of the samples were measured by the Nanodrop ND-2000C Spectrophotometer (Thermo Scientific, Waltham, MA, USA).

### Primers synthesis, polymerase chain reaction, DNA sequencing and genotyping

The promoter region (about 600/700 bp) of the gene encoding the *LALBA* (from about 600 bp upstream of exon 1) has been sequenced in bovine, buffalo, goat and sheep (10 samples per species randomly chosen) for the identification and validation of the SINE element described by Nijman et al [12] as useful marker for genetic traceability within ruminants.

The following primer pair was used for PCR reaction and sequencing: 5′-TTTAGAACTAGTATCCTAAACTCTA-3′ (forward) and 5′-CCAACCACCAATACCACTAA-3′ (reverse). The primers were designed using DNASIS-Pro software (Hitachi, Tokyo, Japan) on conserved regions of the *LALBA* gene promoter. The sequences of cattle (JN258330, nt 261–285 and complementary to nt 980–961), buffalo (NW_005784627, nt 542326–542350 and complementary to nt 543055–543036), goat (NC_030812, nt 30811088–30811112 and complementary to nt 30811880–30811861), and sheep (CP011888.1, nt 137544319–137544343 and complementary to nt 137545188–137545169) were used as templates.

The 50 μL reaction mix included: 100 ng of genomic DNA, 50 mM KCl, 10 mM Tris-HCl (pH 9.0), 0.1% Triton X-100, 1.5 mM MgCl_2_, 10 pmol of each primer, dNTPs each at 200 μM, 1.25 U Taq DNA Polymerase (Promega, Madison, WI, USA), and 0.04% bovine serum albumin. The thermal condition for the amplification consisted of an initial denaturation at 97°C for 2 min, annealing at 61.5°C for 45 s and extension at 72°C for 2 min and 30 s, followed by 30 cycles at 94°C for 45 s, 61.5°C for 45 s, and 72°C for 10 min. A final extension of 10 min was accomplished to end the reaction.

All PCR products were analysed directly by electrophoresis in 3.5% Tris-borate-ethylenediaminetetraacetic acid (TBE) agarose gel (Bio-Rad, Hercules, CA, USA) in 0.5×TBE buffer and stained with SYBR green nucleic acid stain (Lonza Rockland, Inc., Rockland, ME, USA). The PCR products were sequenced on both strands at CEINGE-Biotecnologie Avanzate (Naples, Italy).

For the development of a single PCR protocol that allows the simultaneous identification of DNA from four species of ruminants the following internal primer pair was used: 5′-CAC TGATCTTAAAGCTCAGGTT-3′ (forward) and 5′-TCAGAG TAGGCCACAGAAG-3′ (reverse).

Primers were designed after the comparative analysis of the sequences newly determined at the *LALBA* promoter for the four species investigated. Reaction mix and thermal condition were performed as reported above.

### Sequence analysis and multiple sequence alignment

Pairwise sequence alignments were carried out using NCBI-BLASTN version 2.2.5 and DNAsis pro Software v2.0 (Hitachi, Japan) in combination with manual adjustments.

## RESULTS AND DISCUSSION

**Figure 1:**
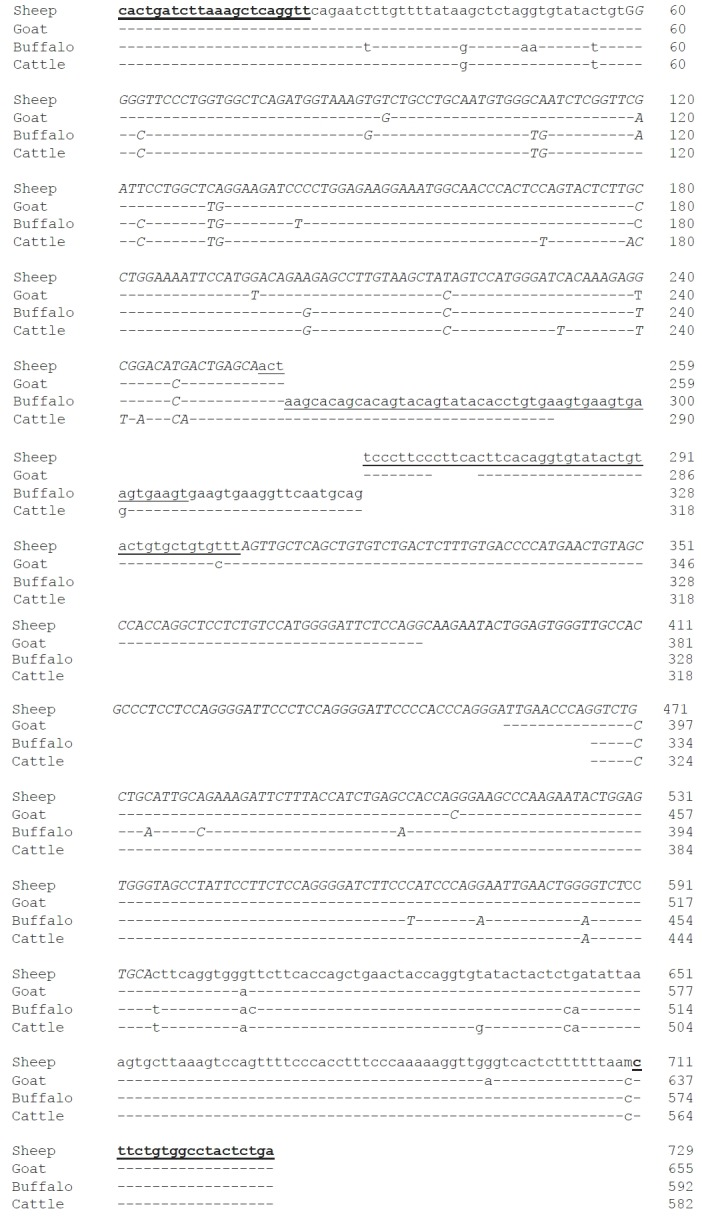
Homology between the nucleotide (nt) sequences of the 5′ flanking region of sheep (upper line), goat, buffalo and cattle α-lactalbumin (*LALBA*) gene. Dashes represent nt identical to those in upper lines. Primers for the simultaneous identification of DNA from four species of ruminants are in shaded bold letters. Artiodactyla retroposons sequences are in uppercase and italic letters and the 49 bp invertion is underlined.

**Figure 2:**
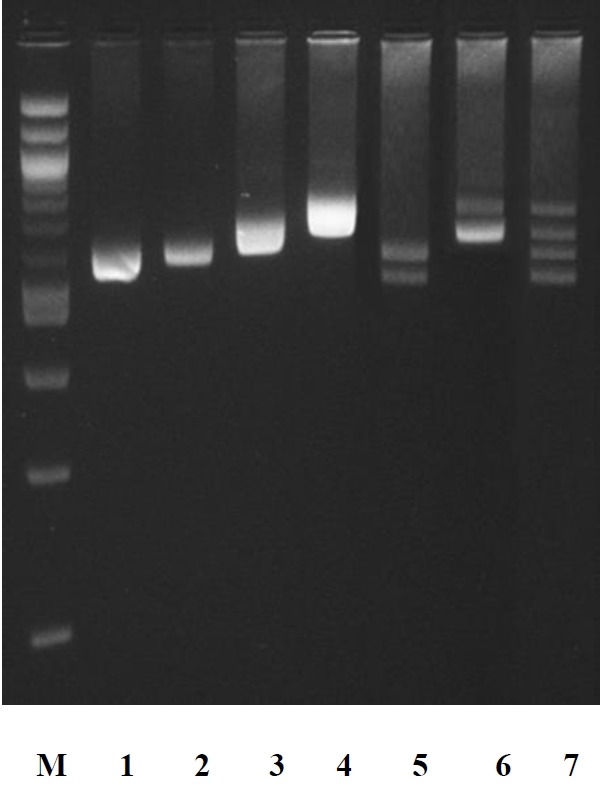
Electrophoretic pattern of amplification products by polymerase chain reaction of DNA region including the 5′-end of the gene coding for bovine (pattern 1), buffalo (pattern 2), goats (pattern 3), and sheep (pattern 4) α-lactalbumin (*LALBA*). Pattern 5: DNA amplification products from buffalo and cattle. Pattern 6: DNA amplification products from sheep and goats. Pattern 7: DNA amplification products from cattle, buffalo, goat and sheep. M: 2-log DNA ladder (0.1 to 10.0 kb) (New England Biolabs, Ipswich, MA, USA).

In ruminants, the amplification of the specific SINE sequence at the *LALBA* DNA promoter [12] is particularly suitable as a tool for the genetic traceability in food products. In fact, it is monomorphic intra-species (buffalo, bovine, goat, and sheep) and variable in size among the species. This allows the identification of species-specific DNA using a single PCR amplification. We verified by PCR screening and sequencing that each species (30 samples per species randomly chosen) gave a specific amplicon, whose sizes were 582 bp in cattle, 592 bp in buffalo, 655 in goat, and 729 bp in sheep (Figures 1, 2).

In detail, sequencing revealed that all ruminants (sheep, goat, cattle, and buffalo) shared one Bov-tA3, followed by two (sheep) or one (cattle and buffalo) adjacent Bov-tA2, whereas one Bov-tA1 and one Bov-tA2 adjacent elements characterize goats. Furthermore, the variable length of the amplification product was also caused by different small truncations in the SINEs and by deletions/insertions in the DNA region between the Bov-tA3 and the downstream elements (Figure 1). In this last tract of DNA, the buffalo and bovine species are characterized by an almost perfect inversion of 49 bp with respect to ovine and caprine species (underlined in Figure 1).

The PCR amplifications of cattle/buffalo, goat/sheep, and cattle/buffalo/sheep/goat DNA mixtures (each at equal concentration) showed electrophoretic amplicon sizes equal to those produced by each individual amplification of the species tested (Figure 2).

It has been also evaluated the sensitivity of the method for identification of the species in mixtures of DNA. For this purpose, a PCR reaction was carried out on a mixture of cattle, buffalo, goat and sheep DNA with concentrations of 0.1, 0.25, 0.5, 1, 5, 10, and 100 (ng/μL) of each species. The weaker electrophoretic band detected by agarose gel corresponded to the percentage of 0.25 ng/μL DNA present in the mixture. Then, PCR products from mixture containing 0.25 ng/μL of DNA were sequenced and the analysis confirmed the amplification of *LALBA* DNA promoter region investigated in sheep, goat, cattle, and buffalo. Therefore, PCR assays reported in this study showed a limit of resolution equal to 0.25 ng/μL. This limit of detection was also reported by Matsunaga et al [15] for the identification of meat species and meat products by PCR assay.

Several researchers have previously reported PCR assays for detection of DNAs of animal origin in feed by primers based on sequences of short or long interspersed repetitive elements [16]. However, there is an important advantage for our intra-SINE-based PCR methods over previously reported approaches. In fact, the proposed method allows distinguishing unambiguously the different species in the context of suborder *Ruminantia* based on PCR products size. Therefore, this method can be useful for a rapid and economic identification of the main domestic ruminant species in foodstuffs for traceability purposes, including milk inspection to guarantee the “Mozzarella di bufala” PDO supply chain from the fraudulent addition of cheaper milk (cattle, goat, and sheep).

## CONCLUSION

In this study, we propose a novel and valuable tool for the detection of ruminant biological components in dairy products. The simplicity of the suggested method; the high sensitivity of the DNA based reaction, which can be performed also for processed dairy products; the rough quantitative assessment based on a simple agarose gel electrophoresis as initial screening tool, gives the opportunity to perform a rapid inspection activity for the detection of fraudulent adulteration of buffalo milk and/or dairy products. Furthermore, this assay format minimizes the cost of analyses on a large scale and gives most laboratories with basic equipment and average economic resources the ability to perform these assays.
